# Determinants of delay in care seeking among children under five with fever in Dodoma region, central Tanzania: a cross-sectional study

**DOI:** 10.1186/1475-2875-13-348

**Published:** 2014-09-03

**Authors:** Telemu Kassile, Razack Lokina, Phares Mujinja, Bruno P Mmbando

**Affiliations:** Faculty of Science, Sokoine University of Agriculture, PO Box 3038, Morogoro, Tanzania; College of Social Sciences, University of Dar es Salaam, PO Box 35045, Dar es Salaam, Tanzania; School of Public Health and Social Sciences, Muhimbili University of Health and Allied Sciences, PO Box 65015, Dar es Salaam, Tanzania; Tanga Research Centre, National Institute for Medical Research, PO Box 5004, Tanga, Tanzania

**Keywords:** Care-seeking behaviour, Delay, Febrile illnesses, Fever, Malaria, Tanzania

## Abstract

**Background:**

Early diagnosis and timely treatment of malaria is recognized as a fundamental element to the control of the disease. Although access to health services in Tanzania is improved, still many people seek medical care when it is too late or not at all. This study aimed to determine factors associated with delay in seeking treatment for fever among children under five in Tanzania.

**Methods:**

A three-stage cluster sampling design was used to sample households with children under five in Dodoma region, central Tanzania between October 2010 and January 2011. Information on illness and health-seeking behaviours in the previous four weeks was collected using a structured questionnaire. A multivariable logistic regression was used to investigate determinants of delay in treatment-seeking behaviour while accounting for sample design.

**Results:**

A total of 287 under-five children with fever whose caretakers sought medical care were involved in the study. Of these, 55.4% were taken for medical care after 24 hours of onset of fever. The median time of delay in fever care seeking was two days. Children who lived with both biological parents were less likely to be delayed for medical care compared to those with either one or both of their biological parents absent from home (OR = 0.42, 95% CI: 0.24, 0.74). Children from households with two to three under-five children were more likely to be delayed for medical care compared to children from households with only one child (OR = 1.54, 95% CI: 1.04, 2.26). Also, children living in a distance ≥5 kilometres from the nearest health facility were about twice (95% CI: 1.11, 2.72) as likely to delay to be taken for medical care than those in the shorter distances.

**Conclusion:**

Living with non-biological parents, high number of under-fives in household, and long distance to the nearest health facility were important factors for delay in seeking healthcare. Programmes to improve education on equity in social services, family planning, and access to health facilities are required for better healthcare and development of children.

## Background

Fever is one of the major markers of an illness [1] and one of the frequently reported causes of under-five children’s caretakers visits to healthcare facilities [2, 3]. It has been shown that caretakers generally have a good biomedical understanding of febrile illnesses in terms of both types and symptoms [4–6]. In most cases, caretakers perceive fever as malaria. In all types of malaria that are perceived by caretakers, fever is the most commonly mentioned symptom [7]. In Tanzania, malaria accounts for over 30% of the national disease burden [8]. It is responsible for most cases of morbidity and mortality especially among children aged under five years [9].

The World Health Organization recognizes that early diagnosis and prompt treatment, within 24 hours of onset of symptoms, is an essential element of malaria control [10]. This is primarily because early medical care reduces the chance of progression of the illness to severe disease [11]. Studies have indicated that home management of febrile illness by community health workers and through drug sellers improves timely access to treatment and has been associated with reduction of malaria infection [12–14]. Sharma [15] noted that timely and appropriate treatment preferably within 24 hours of onset of illness symptoms resulted into reduced severe morbidity and probability of mortality among children under the age of five years. In spite of this, evidence shows that most malaria-related deaths in malaria-affected countries occur at home without receiving appropriate medical care, and when care is sought, it is often delayed [16]. Wiseman and colleagues [17] observed that a considerable proportion of deaths among under-five children in sub-Saharan Africa occur in part because of delays in seeking medical care.

Empirical evidence suggests that early treatment-seeking behaviour is influenced by numerous factors. A study on cancer patients indicated that more educated patients have similar patterns of delay to those of less educated patients [18]. On the other hand, studies in malaria-endemic areas have shown that caretakers with low level of education were more likely to delay in seeking malaria treatment for children [19, 20]. Greenwald and colleagues [21] argue that failure of individuals to acknowledge that something is wrong or vulnerable to a disease may result into delay in presentation to a healthcare provider. Focusing on patients diagnosed with cancer disease, the authors [21] found that previous experience of use of healthcare facilities and occupation status of the patient were responsible factors for most variation in observed delays.

Caretakers often identify malaria as fever along with other symptoms or signs [22]. Studies have observed that caretakers perceive fever differently, mostly as a mild [23] or as a normal [4] disease. Even in situations in which the cause of the fever was believed to be malaria, caretakers still perceived their children’s illness as mild [24]. It is recognized that interventions which increase individuals’ participation in healthcare and which promote greater knowledge of symptoms and outcomes will facilitate proper healthcare seeking and services utilization [25]. Nonetheless, previous studies, for example [4, 22, 26] on delay to seek healthcare for under-five children have considered the impact of the demand-side, the supply-side, or both determinants of delay on a specific child’s febrile illness, largely ignoring the influence of other symptoms or signs on the decision when to seek healthcare for a particular symptom of interest. Studies have shown for example, that factors including ease of access, satisfaction [27] as well as cost [28] of services are associated with delay to seek care. Perception that fever/malaria is a normal disease has also been observed to contribute to delay [4]. The impact of the demand-side, supply-side, or both barriers to prompt care for fever when integrated with other symptoms or signs of malaria in under-five children is not well examined. Explaining the role of demand or supply-side determinants of delay for fever while integrating with other febrile illnesses in under-five children may strengthen interventions that aim at promoting timely diagnosis and treatment for childhood malaria.

In an attempt to improve health outcomes, especially of vulnerable groups, Tanzania provides free health services for children under five and pregnant women in all public health facilities [23, 29]. Nevertheless, statistics show that although access to health services is improved, many people still do not seek medical care or do so when it is too late [30]. It is acknowledged that in order to permit the designing of a well-informed intervention strategy, both the context in which the behaviour takes place and the relative weight of the various factors that trigger actions within that context are essential [31]. In the context of Tanzania, there is a shortage of evidence as to what determines delayed care seeking for an illness episode of a suspected malaria infection among children under five. This paper examines determinants of delay in care-seeking behaviour in under-five children with fever from Dodoma region in central Tanzania.

## Methods

### Study site

This cross-sectional study was conducted in Dodoma region, central Tanzania. By 2006, Dodoma region had 268 health facilities, 208 (77.6%) public owned, 16 (6.0%) private, 32 (11.9%) faith based organizations, and 12 (4.5%) under parastatal organizations [32]. Of the total health facilities, 240 (89%) were dispensaries, 21 (8%) health centres, and 7 (2.6%) hospitals. The under-five mortality rate in the region was estimated to be 182.8 in year 2002 [33].

Malaria is the leading cause of morbidity in both the outpatient and inpatient departments in Dodoma region. However, relative to other regions, Dodoma features poorly in many aspects of health. In 2006 for example, statistics from health facilities showed that admissions due to malaria contributed to 62.0% of all health facility admissions, compared to 40.2% of the entire Mainland Tanzania. In the same year, deaths attributed to malaria in health facilities were 54.0% compared to 33.9% of the entire nation [30]. The 2007-08 Tanzania HIV/AIDS and Malaria Indicator Survey indicated a slightly higher prevalence of fever (19.5%) in children under five compared to 19.0% in the Mainland Tanzania [34]. Nevertheless, the proportion of children 6-59 months who tested positive for malaria was 12.8% against 18.1% of the entire nation. Artemisinin-based combination therapy, the first-line treatment in Tanzania, is the most used anti-malarial drug in all health facilities [34]. The most common malaria species in the region is *Plasmodium falciparum* [35].

### Sampling procedure

The desired target population for the study were all under-five children in the sampled administrative units. Households with at least one child under the age five were eligible for the sampling. A household was defined as a person or group of persons (whether related or not) who live together and share the same food bowl, while a household member was defined as any person (including domestic helpers) living in the same house and sharing meals and information [36]. In order to arrive at the household level, a three-stage cluster sampling procedure was employed. The primary sampling unit was the district in the region, the secondary sampling unit was the village for the case of rural setting or street for the case of urban setting, and the tertiary sampling unit was the household. Districts and villages or streets were selected proportional to size using the cumulative total method sampling technique while households in the selected villages or streets were selected by simple random sampling [37].

### Sample size

Calculation of the sample size was based on the key indicator of the study, the desired precision, design effect, average household size, non-response rate, and an estimate of the percentage of the total population accounted for by the target population and for which the key indicator was based. The key indicator that the study aimed to estimate was the likelihood that a case of malaria (proxied by fever) in under-five children was expected to be reported by the primary caretakers (mothers or guardians) in the sampled households. The probability of reporting a case of malaria in the study was estimated with a 5% margin of relative error at the 95% level of confidence. The resulting estimated sample size for the study was 1,073 households. The calculated sample size was rounded up to 1,080 households with at least one child under the age five. The sample was obtained from four districts of Dodoma Urban, Bahi, Kondoa, and Mpwapwa, out of six districts which were officially recognized at the time of designing this study. In each selected district, 18 villages or streets were sampled, resulting into a total of 72 villages or streets. Moreover, in each selected village or street, a sample of 15 households with children under the age of five was selected through simple random sampling. Due to non-response, only 1,027 out of 1,080 (95%) households were successfully interviewed. However, the analysis in this paper uses only a sub-sample of the data for which fever was reported in under-five children and for which medical care for the symptom was sought by the caretakers.

### Data collection

The data were collected by trained research assistants between October 2010 and January 2011. Prior to the data collection process, the research assistants had a four-day training on various aspects of the study, including the purpose of the study and data collection method. Data were collected through face-to-face interviews using a structured questionnaire, which consisted of items adapted from validated questionnaires (including the Tanzania Demographic and Health Survey, Living Standards Measurement Study and Tanzania HIV/AIDS Malaria Indicator Survey). The questionnaire was designed in English, and translated into Kiswahili (a widely spoken language in Tanzania) to facilitate the interviews. In order to ensure that the original meanings of the various items of the questionnaire were maintained, the Kiswahili version was sent to an independent researcher conversant in both English and Kiswahili to translate it back into English. The two versions were scrutinized systematically to identify any discrepancies in wording or sentence formulation. Inconsistencies were checked and synchronized accordingly. The final Kiswahili version was pre-tested to a sample of 30 households from a ward outside the study area, which consisted of both rural and urban characteristics in Dodoma Municipality. Pre-testing was aimed to check for: (i) wording of the questions, i.e., whether the questions were understandable to the respondents; (ii) plausible response categories of questions; and (iii) sequence of questions in the questionnaire. Some measure items such as household expenditure on medical and non-medical items were found to be not feasible to yield reliable responses, thus were dropped in the final version of the questionnaire. The questionnaire covered household characteristics such as age, sex, education and occupation of caretaker and that of head of household, possession of household-owned assets, housing structure and materials, main source of power for cooking and lighting, and household size; and community characteristics such as approximate distance (in kilometres) to the nearest health facility and marketplace. Information on distance was obtained from local leaders and people in the communities who were knowledgeable about approximate distance to the nearest health facility or marketplace. The child characteristics collected included age (in months), sex and biological relationship with the head of household. Furthermore, information on illness and health-seeking behaviours for each child was collected. Caretakers were asked whether the child experienced an episode of fever within four weeks preceding the interview and how they perceived it (its severity). Also, information on the number of days the child was ill with fever and the course of action that was taken was explored. Apart from fever, information on convulsion, diarrhoea, cough/flu and vomiting episodes were collected.

Interviews were conducted at the respondents’ households. It was heads of households or their spouses who provided information on socioeconomic and demographic characteristics of households. Where the head of the household or spouse was not available, a knowledgeable adult respondent among the members of the household was interviewed. On the other hand, respondents on matters of child health were the primary caretakers (preferably women). The study restricted to women respondents in matters of child health because they are often the primary caretakers of under-five children. Therefore, they stand a better chance of providing health-related information including assessment of illnesses, their severity and information on treatment for their children [38]. In matters related to child health, whenever the respondent was provisionally unavailable to be interviewed at the first visit of the household, up to two additional visits were made. No substitution was made for those who were either unavailable or unwilling to participate in the study.

### Research clearance and ethical considerations

The study was approved by the Department of Economics of the College of Social Sciences at the University of Dar es Salaam. The permission to carry out research in the region was obtained from the Regional Administrative Secretary of Dodoma region, and from district executive directors (DED) of the four districts. DEDs informed lower level administrative authorities about the study. At the household level, preceding the interviews, respondents who freely chose to participate in the study gave oral informed consent through a consent statement, which was read out to each respondent.

### Data management

The collected data were entered in the Statistical Package for the Social Sciences (SPSS) for Windows version 16.0 software. In order to verify the precision of data entry in SPSS, two generic data verification strategies were employed after the data entry. First, 10% randomly selected questionnaires were thoroughly checked. Secondly, descriptive statistics and frequency distributions of each variable were examined. Then, cross-tabulations to search for additional data entry problems were also carried out.

### Data analysis

The total time elapsed between the onset of fever to the time of seeking treatment was defined as early (coded as 0) if care was sought within 24 hours (within one day inclusive) after the onset of fever, and delayed (coded as 1) if care was sought after 24 hours of the onset of fever [11, 17]. To investigate the factors that are associated with delay to seek medical care a binary choice model derived from the assumed relationship between caretakers’ underlying propensity to seek delayed care and a set of explanatory variables was used. Therefore, the relationship between observed delay and the explanatory variables is modelled as a logistic regression in which Pr(*y* = 1) = exp(*β*′*x*)/1 + exp(*β*′*x*). The analysis was done while accounting for sample design (unequal probability of sampling and clustering) using a survey logistic procedure (SURVEYLOGISTIC) in which odds ratios (ORs) and 95% confidence intervals (CIs) were estimated in the SAS system version 9.2 (SAS Institute, Inc, Cary, NC, USA). The cluster statement, using district as clustering variable, was used to take into account correlations among observations from the same district.

Descriptive statistics were also used to describe the characteristics of the data. For continuous variables, mean and standard errors (SEs) were calculated while for categorical variables, frequencies and percentages were calculated. For skewed variables, the median was used instead of the mean. The procedures SURVEYMEANS and SURVEYFREQ were used to account for sample design in computing the means together with their corresponding SEs, and frequencies together with their corresponding percentages, respectively. Variables whose impacts on delay to seek medical care were assessed in this paper are given in Table 1. The wealth indicator variable to proxy for household economic status was created using household-owned assets and housing characteristics. This was achieved using the principle component analysis technique [39, 40]. Consistent with studies such as demographic and health surveys, households were divided into socio-economic quintiles (poorest, second, middle, fourth, highest) based on the factor scores in the first principal component.

**Table 1:** **Children whose caretakers sought early or delayed treatment (** ***n*** **= 287)**

Characteristic	Sought early or delayed medical care ^1^		
	Early 132 (44.6%)	Delayed 155 (55.4%)	All children (***n*** = 287)
Age (in months) of child:			
0-11	30 (10.6)	23 (8.5)	53 (19.0)
12-23	36 (12.8)	50 (17.7)	86 (30.5)
24-35	22 (7.0)	32 (11.4)	54 (18.3)
36-47	26 (8.6)	29 (10.2)	55 (18.8)
48-59	18 (5.6)	21 (7.7)	39 (13.3)
Sex of child (male)	68 (23.2)	81 (29.2)	149 (52.4)
Relationship of child to head of household (non-biological son/daughter)	7 (2.3)	17 (5.8)	24 (8.1)
Child had convulsion (yes)	6 (2.0)	6 (2.2)	12 (4.1)
Child had diarrhoea (yes)	65 (22.6)	67 (23.9)	132 (46.5)
Child had cough/flu (yes)	96 (33.0)	109 (40.2)	205 (73.3)
Child had vomiting (yes)	47 (15.4)	45 (16.1)	92 (31.4)
Fever perceived as a sign of a disease (yes)	103 (34.2)	111 (39.4)	214 (73.6)
Perceived severity of fever for child:			
Mild	57 (19.5)	81 (28.6)	138 (48.0)
Moderate	58 (19.2)	50 (17.9)	108 (37.0)
Severe	17 (5.9)	24 (9.0)	41 (14.9)
Both biological parents stay at home (no)	30 (10.2)	51 (17.5)	81 (27.7)
Biological mother stays at home (no)	3 (0.9)	4 (1.5)	7 (2.3)
Number of under-five children in household (2-3)	24 (8.2)	37 (13.1)	61 (21.4)
*Caretakers*			
Age (years), mean (SE)	30.0 (0.4)	29.8 (0.5)	29.9 (0.3)
Education:			
No education	27 (9.2)	36 (13.7)	63 (22.8)
Primary	98 (33.2)	110 (38.9)	208 (72.1)
Secondary and above	7 (2.2)	9 (2.8)	16 (5.0)
Occupation:			
Unemployed	16 (4.4)	6 (1.6)	22 (6.0)
Agricultural activities	90 (31.4)	117 (44.0)	207 (75.4)
Non-agricultural activities	26 (8.7)	32 (10.0)	58 (18.5)
Years in current place of residence (always)	67 (23.3)	80 (28.8)	147 (52.0)
Marital status (married)	106 (35.4)	109 (39.7)	215 (75.2)
Main source of information in matters of health (health worker)	104 (35.5)	121 (43.3)	225 (78.8)
*Head of household*			
Sex (male)	108 (36.2)	116 (42.1)	224 (78.2)
Age (years), mean (SE)	36.5 (2.3)	37.6 (1.6)	37.1 (1.4)
Education:			
No education	23 (8.4)	33 (12.2)	56 (20.7)
Primary	98 (32.7)	114 (40.9)	212 (73.6)
Secondary and above	11 (3.4)	8 (2.3)	19 (5.7)
Occupation:			
Unemployed	4 (1.5)	4 (1.1)	8 (2.6)
Agricultural activities	98 (35.0)	133 (49.4)	231 (84.4)
Non-agricultural activities	30 (8.1)	18 (4.9)	48 (13.0)
Household size, mean (SE)	5.1 (0.4)	5.3 (0.2)	5.2 (0.2)
Economic status:			
Poorest	28 (10.0)	40 (14.8)	68 (24.8)
Second	31 (10.9)	49 (17.4)	80 (28.2)
Middle	25 (9.7)	32 (12.5)	57 (22.1)
Fourth	30 (9.9)	24 (8.3)	54 (18.2)
Highest	18 (4.2)	10 (2.4)	28 (6.6)
Place of residence (rural)	98 (34.9)	139 (51.6)	237 (86.5)
Distance to nearest HF (≥5 km)	71 (24.3)	107 (39.6)	178 (63.9)
Distance to nearest market (≥5 km)	81 (28.7)	124 (46.0)	205 (74.7)
Main road passable throughout the year (no)	37 (13.5)	40 (14.6)	77 (28.1)
Perceived quality of main road (poor)	54 (19.4)	61 (22.4)	115 (41.8)

^1^Data are presented as frequency (%) or mean (SE) for categorical and continuous variables, respectively.

In order to obtain the most parsimonious model, potential explanatory variables for use in the multivariable logistic regression analysis through the SURVEYLOGISTIC procedure were selected using the stepwise variable selection procedure. This was achieved using the standard logistic procedure. A significance level of 0.3 was chosen to allow a variable into the model while a significance level of 0.35 was chosen to allow a variable stay into the model. These arbitrary levels of significance were considered appropriate against the traditional levels such as 0.05 since the later may fail to identify key explanatory variables [41]. All results are based on the weighted data. A *p-*value < 0.05 was considered significant.

## Results

### Descriptive statistics

**Figure 1:**
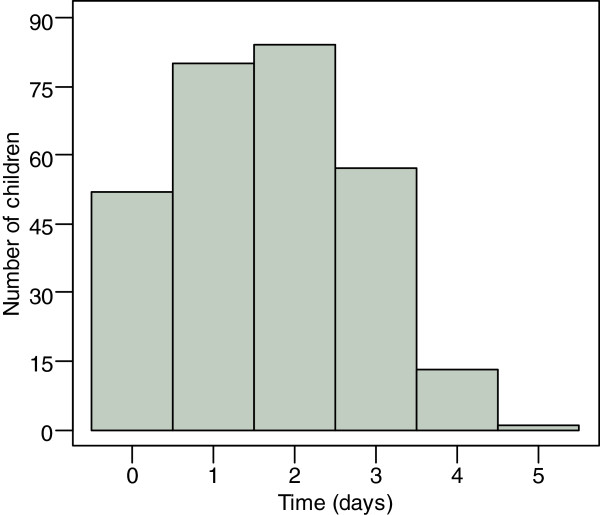
**Days elapsed before medical care was first sought.**

A total of 1,390 under-five children aged between 0-59 months were found from 1,027 successfully interviewed households. Of the total households, 692 (67.4%) had one eligible child under the age of five while 307 (29.9%) and 28 (2.7%) of the households had two and three children, respectively. Of the total children in the study, 329 (23.7%) had fever within the previous four weeks and the majority of them 287 (87.2%) had medical care sought for the symptom. Unless stated, the results will concentrate on the latter. For this subset of the data, 226 households had one eligible child, 29 households had two eligible children, while only one household had three eligible children. Figure 1 displays the distribution of days that elapsed before the caretaker sought medical care for fever. Fifty-two children (16.8%) with fever were taken for medical care on the same day of the symptom onset. The number of children increased, reaching 80 (27.7%) and 84 (29.6%) after one day and two days of the symptom, respectively, but declined afterwards, reaching 57 (20.7%) and 13 (4.8%) after three and four days of the symptom, respectively. Only one child (0.4%) was taken for medical care after five days of recognizing the symptom (Figure 1). The median duration of fever was seven days with a range of one to 28 days. On the other hand, the median time of delay in fever care seeking was two days. Also, the findings reveal that most of the children in rural areas (69.9%) were from households located at a distance of at least 5 kilometres from the nearest health facility compared to 22.2% of children in urban areas.

Table 1 presents summary statistics of children disaggregated by time (early or delay). A total of 132 children (44.6%) received early medical care. The majority of the children, 206 (72.3%) were living with both of their biological parents, while 280 (97.7%) were living with their biological mothers. Caretakers of 63 (22.8%) of the children had no education, 16 (5.0%) had at least secondary education while caretakers of the majority of children, 208 (72.1%) had primary education level. Caretakers of most of the children, 215 (75.2%) and 207 (75.4%) were married and employed in agricultural-related activities, respectively. Caretakers of 58 (18.5%) and of few, 22 (6.0%) of the children were employed in non-agricultural activities and unemployed, respectively (Table 1).

### Determinants of delay

Table 2 presents results of modelling the probability of delay for medical care. Age of child appears to be a significant determinant of delay to seek treatment for fever. However, the effect of age on delays was not consistent across all age groups. The only statistically significant difference was between infants and children in the age group of 23-35 months. Children in age group 23-35 were 2.3 times more likely (95% CI: 1.10, 4.83) to delay seeking medical care than infants, adjusting for other variables. All other age groups had slightly longer delays in seeking healthcare relative to infants; however, the differences were not statistically significant. Children who had diarrhoea were 26% less likely (95% CI: 16%-36%) to delay to be taken for medical care than children who had no diarrhoea, adjusting for other variables. Children who had both biological mother and father at home were less likely to be delayed for medical care compared to those who did not have either of the two or both biological parents, adjusting for other variables (OR = 0.42, 95% CI: 0.24, 0.74).

**Table 2:** **Unadjusted and adjusted ORs and 95% CIs of delay in fever care-seeking behaviour for under-five children (** ***n*** **= 287)**

Variable	Unadjusted OR (95% CI)	Adjusted ^1^OR (95% CI)
Age group of child (months):		
0-11	1.00	1.00
12-23	1.72 (0.86, 3.41)	1.97 (0.87, 4.50)
24-35	2.02 (0.90, 4.54)	2.30 (1.10, 4.83)
36-47	1.48 (0.47, 4.64)	1.38 (0.38, 5.03)
48-59	1.72 (0.61, 4.83)	1.84 (0.48, 7.04)
Child had convulsion (yes)	0.88 (0.30, 2.55)	0.93 (0.23, 3.80)
Child had diarrhoea (yes)	0.73 (0.61, 0.87)	0.74 (0.64, 0.84)
Child had cough/flu (yes)	0.92 (0.65, 1.31)	0.79 (0.49, 1.27)
Child had vomiting (yes)	0.78 (0.60, 1.00)	0.91 (0.63, 1.32)
Both biological parents stay at home (yes)	0.64 (0.35, 1.16)	0.42 (0.24, 0.74)
Number of under-five children in household (2-3)	1.36 (0.93, 2.01)	1.54 (1.04, 2.26)
Perceived severity of fever for child:		
Mild	1.00	1.00
Moderate	0.64 (0.40, 1.00)	0.74 (0.40, 1.35)
Severe	1.05 (0.41, 2.69)	1.30 (0.55, 3.04)
Place of residence (rural)	3.68 (1.23, 11.01)	3.09 (0.90, 10.66)
Distance to nearest health facility (≥5 km)	2.09 (0.99, 4.38)	1.74 (1.11, 2.72)
Distance to nearest marketplace (≥5 km)	2.70 (1.08, 6.78)	1.80 (0.78, 4.17)
Perceived quality of main road (poor)	1.14 (0.43, 3.01)	1.71 (0.62, 4.72)

^1^Multivariate logistic regression adjusting for all covariates given in the table.

Children from households in which there were two to three under-five children were more likely to be taken for medical care later than those from households in which there was only one under-five, adjusting for other variables (OR = 1.54, 95% CI: 1.04, 2.26). Distance to the nearest health facility was also a significant predictor of delay. Children in areas where the distance to the nearest health facility was at least 5 kilometres away from the household were nearly two times more likely (95% CI: 1.11, 2.72) to delay to be taken for medical care than those from shorter distances, adjusting for other variables.

## Discussion

A total of 287 under-five children with fever in the four weeks preceding the date of the survey were studied to establish the pattern and determinants of treatment-seeking behaviour of the caretakers. The majority of the children received medical care after two days of onset of symptom. Less than half of the children were taken for medical care early as recommended by the Abuja target of treating malaria within 24 hours of the onset of symptoms [10]. The finding in this study compares with studies in other countries, which show that a significant proportion of under-five children receive treatment after 24 hours. For example, a study in Ghana found that 33% of under-five children suspected of being infected with malaria received treatment within 48 hours of symptom recognition, while only 11% received treatment within 24 hours [42]. Similar findings were reported in Myanmar, 32.0% [43] and in Nigeria, 22% [19]. In Tanzania, caretakers in Dar es Salaam waited for at least 48 hours after onset of fever before the decision to take a sick under-five to a health provider was made [44].

The findings in this study have revealed that caretakers sought treatment early for the youngest children (infants) compared to older children and for those with diarrhoea. These results are similar to those reported in Kenya where healthcare seeking was more frequently among children in the age group 0-11 months and those with diarrhoea symptom [45]. Similar findings were reported in urban Dhaka, Bangladesh, where febrile infants were more likely to receive quality care than older children [46].

In the present study, presence at home of both biological parents is significantly associated with prompt care-seeking decision than is the case when one or both biological parents are not at home. This could be due to multiple reasons, including lack of collective decision-making and adequate resources to meet treatment-associated costs such as transport especially in areas where the nearest health facility is far from the household. Chuma and colleagues [47] found that seasonality of income sources and transport costs were among the barriers to access prompt and effective malaria treatment in Kenya. The effect of this limitation is likely to be more pronounced in a single parent environment than in a two-parent milieu. Meanwhile, literature on intra-household allocation of resources shows that household resources for investment in human capital such as health are allocated with a view to maximizing a particular utility function [48]. The utility function is defined over a given set of goods and services subject to a set of constraints, including the household budget. In situations of restricted resources and particularly when the head of household is a non-biological parent of the sick child, the concerned child may fail to receive due attention, such as timely medical care.

The results in this study have shown that children from households in which there were two to three under-five children were more likely to receive medical care late than those from households which had only one under-five. This finding is not surprising in view of the fact that under-five children is a group that mostly requires the attention of primary caretakers within the household. In situations of more than one under-five, when one or all experience an illness it might be difficult to manage all of them at the same time than it is the case when there is only one child in the household. The finding in this study is similar to those reported in other settings. For example, a study conducted in Pakistan [49] found that the number of children in the family was a significant determining factor of the decision to seek healthcare.

In the present study, distance to the nearest health facility is one of the important determining factors of delay in fever care-seeking behaviour for under-five children. The findings in the present study are consistent with studies in many different contexts. For example, with respect to distance to the nearest health facility, a study in Uganda found that shorter distances were associated with timely treatment seeking [50]. Moreover, children from the lowest socio-economic strata were less likely to be taken to a health facility timely. This is not unexpected because long distances to health facilities may require out-of-pocket money to cover transport costs to and from the health facility. In a study to examine determinants of delay in malaria treatment-seeking behaviour for under-five children in south-west Ethiopia, it was shown that children of caretakers who had difficulties to meet transport costs were more likely to receive delayed malaria treatment [51]. In Tanzania, because of existence of many competing needs for the same limited resources, particularly in rural areas [52], caretakers may be persuaded to wait to see if the symptoms will disappear without taking the child to a health facility. This may result into a delayed treatment-seeking decision. Equally, because of limited resources, an individual caretaker may fail to take timely treatment-seeking decision for her perceived sick child because she might be forced to first attend to income-generating activities in order to meet day-to-day basic needs, including food for the household.

A number of studies have reported an association between socioeconomic status-related characteristics and healthcare-seeking behaviour [50, 53–55]. In the present study, household wealth status for example, was not associated with delay to seek medical care. This could be because a large part of the sample in this study was from rural areas (Table 1). As a result, differences in wealth status between groups of individuals might not have been large enough to yield a significant wealth effect.

A comparison between the adjusted and unadjusted results revealed existence of variations in terms of significant variables. For example, the effect of distance to the nearest health facility was found to be insignificant in the unadjusted results in Table 2. Moreover, while there appears to be no significant differences in delay between children from rural areas and those from urban areas based on the adjusted ORs, a significant difference between the two groups is depicted in the unadjusted ORs. Distance to the nearest market place also seems to be significant in the unadjusted ORs, but the same is not significant in the adjusted results. Presence at home of both biological mothers and number of children under the age of five in the household which were significant in the multivariate results, adjusting for the other variables appear to be insignificant in the unadjusted results. Overall, with the exception of the effect of diarrhoea, which is significant in both the adjusted and unadjusted, all the other variables that are significant in the former appear insignificant in the latter.

## Conclusion

The majority of caretakers in the study area seek medical care late. The analysis in this study shows that absence at home of at least one of biological parents, number of under-five children in the household and distance to the nearest health facility predict delayed treatment-seeking decision for fever. Therefore, effective programmes that aim at improving outcomes for febrile under-five children through timely treatment-seeking behaviour should consider reducing distances to health facilities. Meanwhile, programmes to improve education on equity in social services to all children and family planning are necessary for better healthcare and development of under-fives and households at large.
